# Tick-Pathogen Ensembles: Do Molecular Interactions Lead Ecological Innovation?

**DOI:** 10.3389/fcimb.2017.00074

**Published:** 2017-03-13

**Authors:** Alejandro Cabezas-Cruz, Agustín Estrada-Peña, Ryan O. M. Rego, José De la Fuente

**Affiliations:** ^1^UMR BIPAR, Animal Health Laboratory, ANSES, Institut National de la Recherche Agronomique, ENVAMaisons Alfort, France; ^2^Department of Parasitology, Faculty of Science, University of South BohemiaČeské Budějovice, Czechia; ^3^Biology Center, Institute of Parasitology, Czech Academy of SciencesČeské Budějovice, Czechia; ^4^Faculty of Veterinary Medicine, University of ZaragozaZaragoza, Spain; ^5^SaBio. Instituto de Investigación en Recursos Cinegéticos IREC (CSIC-UCLM-JCCM)Ciudad Real, Spain; ^6^Department of Veterinary Pathobiology, Center for Veterinary Health Sciences, Oklahoma State UniversityStillwater, OK, USA

**Keywords:** tick-pathogen interactions, transcriptional reprogramming, epigenetics, ecological adaptation, *Anaplasma phagocytophilum*

Ticks are arthropods distributed worldwide that constitute the most important vectors of diseases to animals, and second to mosquitoes regarding pathogens of public health importance. Ticks are remarkably plastic and can colonize diverse ecological niches of the planet, from tropics to polar areas (de la Fuente et al., 2008). In the last decade, the reports of tick-borne pathogens have increased sharply, motivating vigorous research programs that addressed major questions on the epidemiology of tick-borne diseases, vector-host-pathogen interactions, tick ecology, and tick genomics. Notably, the first tick genome was released this year (Gulia-Nuss et al., 2016), opening new possibilities to explore tick-host-pathogen interactions (de la Fuente et al., 2016a). In contrast, the evolutionary and ecological implications of tick-pathogen associations have received comparatively less attention. Herein, we hypothesized that tick-pathogen associations evolved to form “*intimate epigenetic relationships*” similar to those described for *Theileria* spp. and its vertebrate host (Cheeseman and Weitzman, 2015) in which the pathogen induces transcriptional reprogramming in infected ticks. This will ultimately favor pathogen propagation, but will also select for the most suitable ecological adaptations in the tick vector. These phenotypic and genetic changes may have the potential to be transmitted to the next generation of ticks. As a result, the ecological associations between tick, vertebrates, and pathogens would evolve to maximize pathogen circulation in these communities (Estrada-Peña et al., 2015, 2016).

Our hypothesis was based on the following evidences: (i) tick-borne pathogens induce transcriptional reprogramming in infected tick (Ayllón et al., 2015; Villar et al., 2015; Weisheit et al., 2015) and vertebrate cells (Lee et al., 2008; Bouquet et al., 2016); (ii) tick-borne pathogens produce and secrete effector proteins, nucleomodulins, which constitute a family of proteins produced by bacterial pathogens to control host transcription and other nuclear processes (Bierne and Cossart, 2012), that interact with host epigenetic machinery and induce transcriptional reprogramming (Garcia-Garcia et al., 2009a,b; Rennoll-Bankert et al., 2015; Sinclair et al., 2015; Lina et al., 2016), and (iii) tick-pathogen interactions increase tick fitness (Neelakanta et al., 2010; Belova et al., 2012; Herrmann and Gern, 2015; de la Fuente et al., 2016b).

## Tick-borne pathogens induce transcriptional reprogramming in host cells

Several studies using “omics” technologies have revealed that a common pattern in the infection by tick-borne pathogens is the transcriptional reprograming of the host cells. These pathogens include obligate intracellular bacterial such as *Anaplasma phagocytophilum* (Carlyon et al., 2002; Borjesson et al., 2005; Pedra et al., 2005; Sukumaran et al., 2005; Lee et al., 2008; Ayllón et al., 2015) and *Ehrlichia chaffeensis* (Miura and Rikihisa, 2009), the extracellular bacterial pathogen *Borrelia burgdorferi* (Bouquet et al., 2016) and viruses such as TBEV (Weisheit et al., 2015). This transcriptional reprograming not only affect gene expression but also impact protein abundance (Lin et al., 2011; Ayllón et al., 2015). Among the cellular components and processes affected in ticks by pathogen infection are the cytoskeleton, cell immunity, apoptosis, metabolism, and potentially the posttranslational modification of histone tails (Ayllón et al., 2015; Villar et al., 2015; Cabezas-Cruz et al., 2016). Notably, gene expression regulation by tick-borne pathogens occurs in a tissue-specific manner. For example, to establish an infection in ticks, *A. phagocytophilum* inhibits the apoptosis in infected midgut and salivary glands. However, in tick midgut, *A. phagocytophilum* inhibits the apoptosis by upregulating the Janus kinase (JAK)-signaling transducer activator of transcription (JAK-STAT) pathway, but in salivary glands this bacterium down-regulates the expression of porin, which results in the inhibition of cytochrome c release and the intrinsic apoptosis pathway (Ayllón et al., 2015; Alberdi et al., 2016). Taken together, these findings reveal that during evolution tick-borne pathogens have developed specific mechanisms to manipulate gene expression in host cells.

## Molecular messengers of pathogen manipulation

To manipulate gene expression, pathogens activate signaling pathways or hijack the epigenetic machinery of host cells. Both mechanisms have been described during *A. phagocytophilum* infection in ticks. For example, *A. phagocytophilum* infection triggers expression of antimicrobial peptides in salivary glands that control bacterial load. The expression of this family of antimicrobial peptides is mediated by the activation of the JAK-STAT pathway (Liu et al., 2012). It has also been shown that *A. phagocytophilum* induces the activation of the PI3K signaling pathway leading to actin phosphorylation to increase the expression of the gene *salp16* coding for a tick salivary protein crucial for *A. phagocytophilum* survival (Sultana et al., 2010). However, while signaling pathways activation can explain the regulation of some genes, (Sultana et al., 2010; Liu et al., 2012), it does not explain the massive gene regulation induced by *A. phagocytophilum* infection in ticks (Ayllón et al., 2015). In fact, *A. phagocytophilum* induces the differential expression of 8,516 (from 16,083 gene transcripts identified), 5,394 (12,651) and 2,487 (11,105) genes in *Ixodes scapularis* tick nymphs, adult midguts, and salivary gland, respectively (Ayllón et al., 2015; de la Fuente et al., 2016a).

*A. phagocytophilum* produces a family of proteins called nucleomodulins that control host gene expression at the epigenetic level (Sinclair et al., 2015). In particular, the ankyrin repeat effector protein ankyrin A (AnkA) was reported to be secreted by *A. phagocytophilum* through the bacterial type IV secretion system (T4SS) in infected neutrophils (Garcia-Garcia et al., 2009a,b; Rennoll-Bankert et al., 2015). AnkA enters the granulocyte nucleus, binds stretches of AT-rich DNA and alters transcription of antimicrobial defense genes, including down-regulation of *CYBB*, which codes for a NADPH oxidase 2 (Nox2). This enzyme is involved in the production of reactive oxygen species (ROS), which is crucial in the neutrophil immune response against intracellular bacteria. To achieve this regulatory process, AnkA recruits host histone deacetylase 1 (HDAC1) and decreases histone H3 acetylation in infected cells (Garcia-Garcia et al., 2009a,b). This results in chromatin changes that down-regulate the expression of target genes (e.g., *CYBB*). Remarkably, 50 proteins were identified in the genome of *A. phagocytophilum* that may have a function similar to that of AnkA (Sinclair et al., 2015). In addition, genome wide evidence showed that AnkA not only binds to *CYBB* promoter regions, but broadly throughout all chromosomes and correlates with infection-induced differential gene expression (Dumler et al., 2016). Whether *A. phagocytophilum* AnkA is expressed during tick infection is not known. However, it was recently shown that *I. scapularis* has a homolog of the HDAC1 protein that is over-represented in salivary glands in response to *A. phagocytophilum* infection (Cabezas-Cruz et al., 2016). In addition, pharmacological inhibition of tick HDAC1 reduced the load of *A. phagocytophilum* in ISE6 tick cells (Cabezas-Cruz et al., 2016). This result suggests that *A. phagocytophilum* uses similar strategies to manipulate tick and vertebrate host cells (de la Fuente et al., 2016c). The role of *A. phagocytophilum* nucleomodulins during infection provides the molecular basis for specific and genome wide manipulation of host gene expression.

## Tick-pathogen interactions increase tick fitness

Pathogens must overcome many barriers in order to establish an infection in the tick. Increasing tick fitness by pathogen infection so as to survive would be a *win-win strategy* (de la Fuente et al., 2016b). There are remarkable examples in which pathogens manipulate tick protective responses to facilitate infection but preserving tick feeding and vector capacity to guarantee the survival of both the pathogens and ticks. For example, Neelakanta et al. (2010) demonstrated that *I. scapularis* ticks infected with *A. phagocytophilum* show enhanced fitness against freezing injury due to the induced expression of a tick antifreeze glycoprotein. They further showed that improved survival of infected ticks correlated with higher bacterial infection, therefore providing a direct link between pathogen infection and tick fitness in unfavorable ecological conditions. *A. phagocytophilum* may also affect tick questing behavior by increasing the levels of Heat Shock Proteins (HSP), which also prevent blood-feeding stress and desiccation at high temperatures (Busby et al., 2012; Villar et al., 2015). Tick questing behavior is essential to find new hosts and survive in nature. Similarly, *A. phagocytophilum* does not manipulate the levels of Subolesin, a protein involved in the tick innate immune response, because it affects infection, tick feeding, and reproduction (de la Fuente et al., 2016b). In contrast, Porin levels are down-regulated by *A. phagocytophilum* infection as a mechanism to inhibit apoptosis, but without affecting tick fitness (Ayllón et al., 2015; Alberdi et al., 2016; de la Fuente et al., 2016b). These results support that *A. phagocytophilum*-induced transcriptional reprogramming selectively manipulates the expression of tick genes that increase tick fitness and therefore pathogen circulation.

Although similar molecular mechanisms have not been described for *Borrelia* spp. and TBEV infections, they also appear to increase tick fitness (Herrmann and Gern, 2015). *Borrelia* and TBEV-infected *I. scapularis* and *I. persulcatus* ticks were found at higher questing heights when compared to uninfected ticks. Higher questing height increases the chances of a tick to find a larger host that could accommodate more ticks increasing their feeding possibilities, but at the same time exposes ticks to more desiccating conditions (Lefcort and Durden, 1996; Romashchenko et al., 2012). Low relative humidity is detrimental for ticks because they spend their energy reserves quicker than at higher relative humidity (Randolph and Storey, 1999). The fact that *Borrelia* and TBEV-infected ticks choose higher questing height suggests that these pathogens help ticks to survive under dry conditions. In agreement with this hypothesis, *I. ricinus* infected by *B. burgdorferi* move less toward a humid environment and their survival is higher in highly desiccating conditions (Herrmann and Gern, 2010, 2012).

## Tick-borne pathogens have the potential to lead ecological adaptation during tick evolution

**Figure 1:**
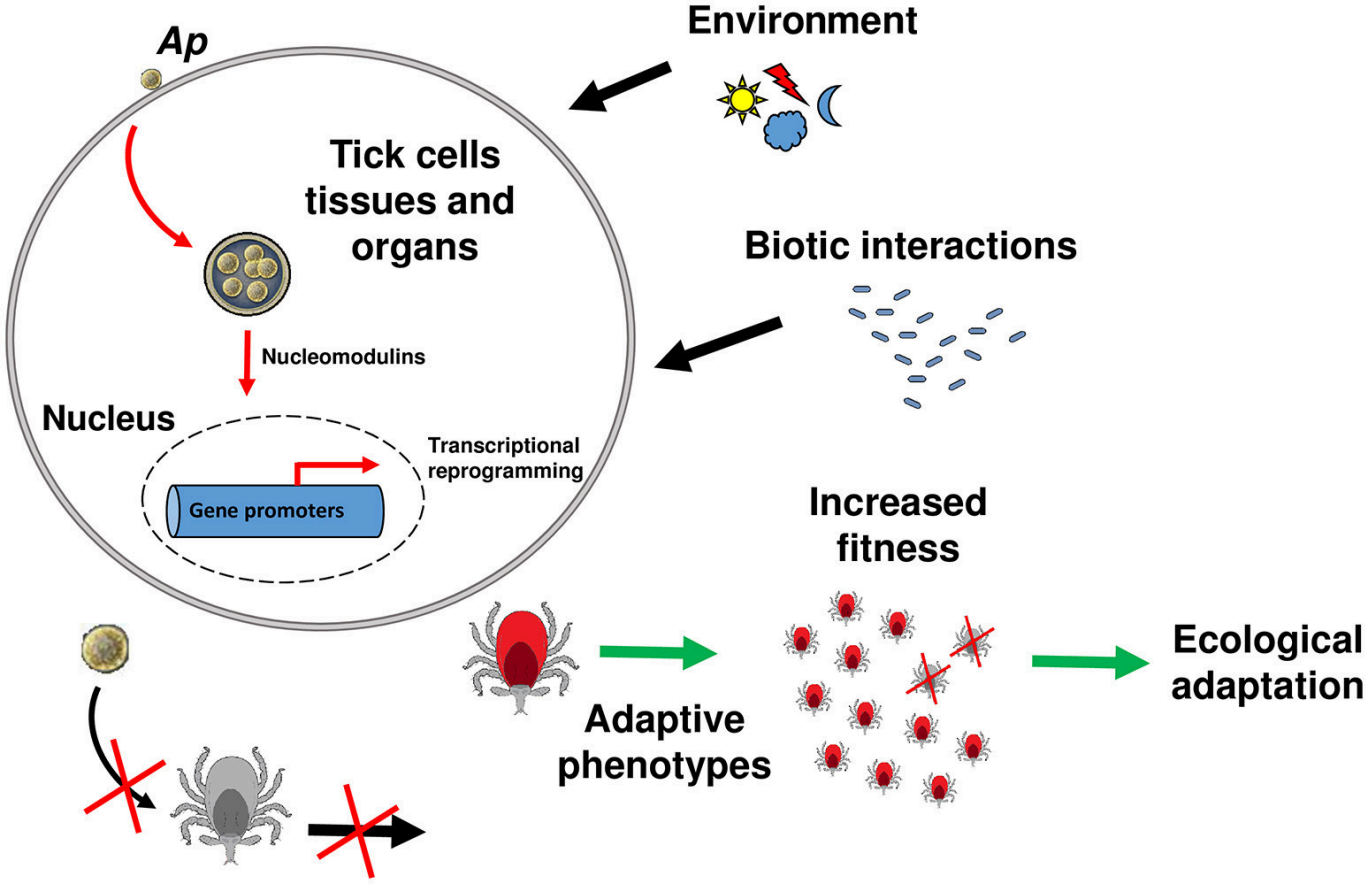
**Model of tick ecological adaptation induced by *A. phagocytophilum***. A model is proposed for the general mechanism of tick manipulation by tick-borne pathogens and induction of ecological adaptation. The intracellular bacterium *A. phagocytophilum* (*Ap*) is used as a model. Upon contact with the host membrane or once inside the parasitophorous vacuole, *A. phagocytophilum* secretes nucleomodulins that will enter the tick cell nucleus and recruit histone modifying enzymes (i.e., HDAC1) to modify the expression of target genes. Some of these genes are involved in traits that favor adaptive phenotypes (red ticks) to abiotic factors (e.g., environmental conditions) or biotic factors (e.g., interactions with microorganisms that may be harmful for the ticks). Histone tail modifications (deacetylation/acetylation, methylation/demethylation, etc) resulting from histone modifying enzymes recruitment, will be passed to the next generation. The ticks able to stablish this “*intimate epigenetic relationships*” (Cheeseman and Weitzman, 2015) with the pathogen will have higher fitness compared to the ticks that are not infected (gray ticks). During evolution, this process will lead to tick ecological adaptation and innovation.

It was generally assumed that DNA changes are the only way information can be passed from parents to the offspring, and that some phenotypic changes acquired during the life span cannot be transmitted to the following generations. Accumulating evidence, however, indicates that both genetic and epigenetic (defined as changes in gene expression due to processes that arise independent of changes in the underlying DNA sequence) have important effects on evolutionary outcomes (Danchin et al., 2011; Gómez-Díaz et al., 2012). While host physiology manipulation by pathogens is a widely accepted phenomenon, we lack evidence of the heritable character of host phenotypes induced by pathogens (Gómez-Díaz et al., 2012; Poulin and Maure, 2015). It was previously proposed that all trans-generational effects on host offspring phenotype that are induced by parasites must involve a strong epigenetic component (Poulin and Thomas, 2008). Given the demonstrated propensity of tick-borne pathogens to modulate tick gene expression, to do so epigenetically, and the increase in tick fitness that can result, we propose that: pathogen-induced effects on tick phenotype have the potential to be transmitted across generations, therefore accelerating the ecological adaptation of ticks to natural environments (Figure 1). The tick antifreeze glycoprotein triggered by *A. phagocytophilum* infection in *I. scapularis* offers a good model to study this phenomenon (Neelakanta et al., 2010). Natural populations of *I. scapularis* in North America inhabit regions where the temperature reaches freezing conditions for much of the winter (Eisen et al., 2016). Ticks infected by *A. phagocytophilum* will be better adapted to cold temperatures in natural environments. It is reasonable to hypothesize that the up-regulation of the antifreeze glycoprotein expression is associated with specific histones or DNA modifications. Inheritance of these epigenetic modifications may transmit the cold-survival phenotype to tick offspring. If this phenotype is advantageous, it may be fixed in the tick population even in the absence of the initial stimulus (i.e., *A. phagocytophilum* infection).

## Author contributions

All authors listed, have made substantial, direct and intellectual contribution to the work, and approved it for publication.

### Conflict of interest statement

The authors declare that the research was conducted in the absence of any commercial or financial relationships that could be construed as a potential conflict of interest.
